# Biopolymers as Sustainable and Active Packaging Materials: Fundamentals and Mechanisms of Antifungal Activities

**DOI:** 10.3390/biom14101224

**Published:** 2024-09-27

**Authors:** Fernanda González-Arancibia, Maribel Mamani, Cristian Valdés, Caterina Contreras-Matté, Eric Pérez, Javier Aguilera, Victoria Rojas, Howard Ramirez-Malule, Rodrigo Andler

**Affiliations:** 1 Escuela de Ingeniería en Biotecnología, Centro de Biotecnología de los Recursos Naturales (Cenbio), Universidad Católica del Maule, Talca 3460000, Chile; 2 Laboratorio de Bioprocesos, Centro de Biotecnología de los Recursos Naturales (Cenbio), Universidad Católica del Maule, Talca 3460000, Chile; 3 Centro de Investigación de Estudios Avanzados del Maule (CIEAM), Vicerrectoría de Investigación y Postgrado, Universidad Católica del Maule, Talca 3460000, Chile; cvaldesv@ucm.cl; 4 Programa de Doctorado en Psicología, Facultad de Ciencias de la Salud, Universidad Católica del Maule, Talca 3460000, Chile; 5 Escuela de Ingeniería Química, Universidad del Valle, Cali A.A. 25360, Colombia

**Keywords:** antifungal, biobased materials, biodegradable packaging, biopolymers

## Abstract

Developing bio-based and biodegradable materials has become important to meet current market demands, government regulations, and environmental concerns. The packaging industry, particularly for food and beverages, is known to be the world’s largest consumer of plastics. Therefore, the demand for sustainable alternatives in this area is needed to meet the industry’s requirements. This review presents the most commonly used bio-based and biodegradable packaging materials, bio-polyesters, and polysaccharide-based polymers. At the same time, a major problem in food packaging is presented: fungal growth and, consequently, food spoilage. Different types of antifungal compounds, both natural and synthetic, are explained in terms of structure and mechanism of action. The main uses of these antifungal compounds and their degree of effectiveness are detailed. State-of-the-art studies have shown a clear trend of increasing studies on incorporating antifungals in biodegradable materials since 2000. The bibliometric networks showed studies on active packaging, biodegradable polymers, films, antimicrobial and antifungal activities, essential oils, starch and polysaccharides, nanocomposites, and nanoparticles. The combination of the development of bio-based and biodegradable materials with the ability to control fungal growth promotes both sustainability and the innovative enhancement of the packaging sector.

## 1. Introduction

The constant growth of the population and the current standard of living have led to an overproduction of plastic materials and, therefore, major disposal problems. Up to 12.7 million metric tons are estimated to be discarded into the oceans yearly [1]. Of the total plastics produced worldwide, 40% is used in packaging [2]. Food packaging, in particular, is of interest for its production volume and environmental impact [3]. However, for adequate food protection, the use of packaging has become essential, and without the current advances in packaging industry technology, the handling of food products would be highly costly and inefficient [4]. Consequently, the development of biodegradable plastic packaging has become necessary as a sustainable material to replace petrochemical plastics [5,6,7]. The term biodegradation is defined as the process of decomposition of organic chemicals by the activity of living organisms or enzymes into harmless products such as CO_2_/CH_4_ and H_2_O [8,9].

For an efficient food transportation process, especially export, it is essential to control postharvest conditions. Fruits and vegetables are very sensitive to deterioration caused by environmental factors, such as temperature, humidity, and plant respiration and transpiration mechanisms [10,11]. In addition, there are other important effects, including loss of firmness, discoloration, and desiccation [12]. Among the different food categories, fruits and vegetables have the highest rate of losses, reaching 66% [13], which makes the development of active packaging for this productive sector interesting.

The handling of fresh foods such as fruits and vegetables in postharvest periods can reach up to 30% losses due to poor chain management and spoilage [14]. Phytopathogenic fungi, which grow postharvest and once mature, can release spores that cause diseases, such as aspergillosis. This disease is produced by fungi of the genus Aspergillus, which can affect both humans and many animals, producing localized non-invasive infections, allergic reactions, and disseminated fatal diseases [15]. This could become a problem for the health and livestock sector. Therefore, it is important to have good food handling during the production process, focusing on the production of functional packaging with antifungal properties. Antifungal properties are understood as the activity of a compound or molecule that destroys or inhibits the growth of fungi.

It is known that controlling respiration and fungal growth is one of the main problems affecting fruits and vegetables during postharvest, especially in long-distance exports. Different fruit and vegetable preservation techniques exist, including electrical, thermal, chemical, and radiation methods [16]. However, many of these methods are expensive, require large amounts of energy, are difficult to control during transport, and can even harm human health [17]. According to Dai et al. [18], physical preservation methods are usually expensive, require special maintenance, and are difficult to play a long-term preservation role in all aspects of the process, while chemical methods often lead to environmental pollution, residues, and hidden hazards, making the chemical preservation method currently hardly reliable for consumers. In recent decades, different sustainable antifungal packaging alternatives have been developed, including the development of materials based on biodegradable polymers with antimicrobial activity and/or the addition of biomolecules or biobased compounds [19]. According to Kahramanoglu et al. [20], preserving fruits and vegetables with biomaterials is the way of the future to guarantee their quality and provide a sustainable solution with a safety seal for human health.

This review is a compilation of the latest trends in biodegradable packaging with antifungal effects or the potential for adding antifungal compounds by different methodologies. A comprehensive description of the composition, activity, and mechanism of action of the main antifungal compounds of natural and synthetic origin is presented.

## 2. Fungal Growth in Fruits

### 2.1. Most Common Phytopathogenic Fungi

Fruit diseases caused by phytopathogenic fungi are responsible for large economic losses. These rot losses are mainly associated with inefficient fruit handling in the postharvest period. This leads to the application of chemicals that control fungal growth, known as fungicides. The indiscriminate and excessive use of different fungicidal agents represents a risk to human and animal health due to food contamination and the accumulation of toxic residues in the environment. As a consequence of market globalization and climate change, this problem is growing at an accelerated rate [21]. Some of the main pathogens of the fungi genera that cause spoilage in food and mainly fruits are *Alternaria*, *Botrytis*, *Lasiodiplodia*, *Penicillium*, *Aspergillus*, *Colletotrichum*, *Fusarium*, *Rhizopus*, and *Mucor* [22]. These are known as the leading causes of the most frequent alterations, especially those related to physical appearance, nutritional value, organoleptic characteristics, conservation difficulty, and allergies and intoxications in consumers. This is because they produce fruiting bodies and spores in harvested fruits that penetrate, invade, and eventually massively colonize the tissue to cause damage and subsequently secrete substances as a result of their secondary metabolism [23].

Some of the most common fungal species that attack fruit production during postharvest produce common symptoms or diseases that, through international food trade, have spread and settled in new regions where they did not exist. An example of the above is *Botrytis cinerea*, a fungus that causes “soft rot” of fruits, which affects the seedling stage to postharvest, including wilting of flowers and rotting of different organs [24]. The fungus *Rhizopus stolonifer* also causes soft rot of fruits and vegetables; it is widely distributed worldwide and appears in fleshy organs of vegetables, flowering plants, and fruits once harvested. *Fusarium semitectum*, a common pathogen in tropical and subtropical countries, is known as a weak fungal parasite responsible for fruit rot [25]. All these fungi are typical postharvest organisms that proliferate on fruits and vegetables. Thus, it is important to control their growth during storage and transport since these are the last stages before sale on the market [26].

### 2.2. Infection Mechanism of Phytopathogenic Fungi

Harvested fruit often develop symptoms of fungal infection during the storage period, when pathogens germinate and penetrate the fruit by breaking the cell wall of plant cells, which is the first defensive barrier against fungi [27]. The state of the fruit will affect the fungal infection process, and some may enter and remain dormant until conditions are appropriate for germination. The ripening phase is ideal for fungal proliferation since the fruit’s defensive system is diminished, the tissue is weak, and in climacteric fruits, the incremental generation of ethylene promotes early ripening [28].

**Figure 1:**
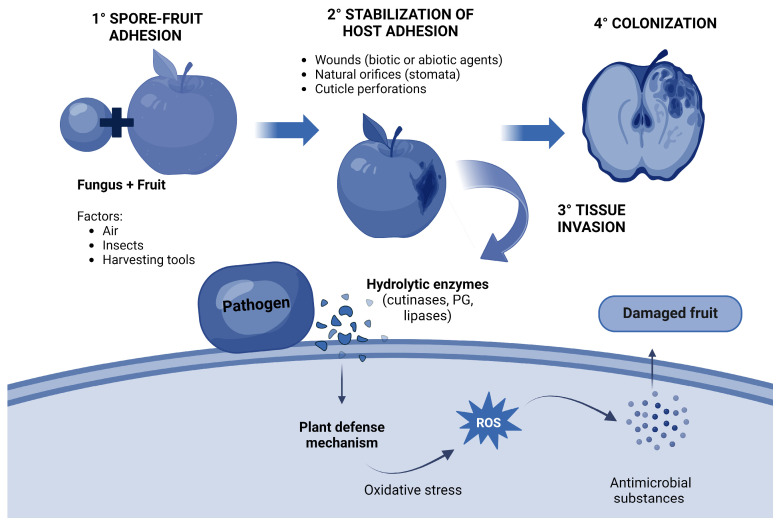
General fungi infection mechanism in fruit.

The infection process consists of four stages: (1) spore-fruit adhesion, (2) stabilization of host adhesion by structures, (3) tissue invasion, and (4) colonization and dissemination. Together, these four stages allow the fungus to enter the fruit, and during this infection process, the fungus produces a variety of metabolites, such as enzymes and toxins, which are adverse to its host. In response, the fruit activates its antifungal defense system [29]. Spores can enter the fruit surface by various factors, including air, insects, and harvesting tools. Meanwhile, the tissue can be infected in three ways: (1) by wounds produced by biotic or abiotic agents, (2) by natural orifices of the plant, or (3) by perforating the fruit cuticle. Once the fungus-host union has occurred, the secretion of hydrolytic enzymes, such as cutinases, polygalacturonases (PG), and lipases, will begin to break down the cell tissue. This initiates the plant’s defense mechanism, resulting in oxidative stress with the production of reactive oxygen species (ROS) and antimicrobial substances [22] (Figure 1). Pathogenic fungi produce toxins that are non-enzymatic compounds that trigger diseases in the host plant [30,31].

Fruit and vegetable spoilage due to fungal infection is and will continue to be a problem. It is important to know about the mechanisms and stages of infection in order to develop intelligent packaging systems to control fungal growth in the postharvest period.

## 3. Bio-Based Packaging Materials

The main function of packaging is to isolate the product from the external environment and protect it from spoilage caused by microorganisms, moisture, gases, dust, odors, and mechanical forces [32]. This is for possible applications in industry, such as developing antimicrobial materials for flexible or rigid food packaging, which has become the focus of researchers and scientists. The interaction between packaging and antimicrobial agents primarily aims to provide a protective barrier against microorganisms and simultaneously reduce the need for chemical preservatives in packaged products.

The biopolymers in this section are divided into two main groups: polyesters and polysaccharides. Although all these polymers are bio-based, not all of them are biodegradable or depend on the scale and complexity of the biodegradation process. While PHA, PCL, cellulose, and starch are compostable in both home and industrial conditions, PLA, PBS, and chitosan are only compostable at the industrial scale, and, in particular, only PHA, cellulose, and starch are completely biodegradable in landfill conditions [33,34,35,36,37].

### 3.1. Polyesters

Polyesters have been widely used in various applications, such as biomedicine, construction, and mainly in packaging. The latter comprises more than 53% of the total bioplastics produced, which, for the year 2019, corresponded to approximately 2.11 million tons worldwide [38]. Different types of polyesters are mainly classified into polymers produced by microorganisms, such as polyhydroxyalkanoate (PHA), and other polymers, such as poly(Ɛ-caprolactone) (PCL), polybutylene succinate (PBS), and polylactic acid (PLA), where microorganisms generate precursors for their synthesis. Biodegradable antimicrobial packaging based on PHA, PCL, PBS, and PLA has been studied in recent years by adding different antifungal, antioxidant, and bactericidal agents (Table 1).

#### 3.1.1. Polyhydroxyalkanoates

**Figure 2:**
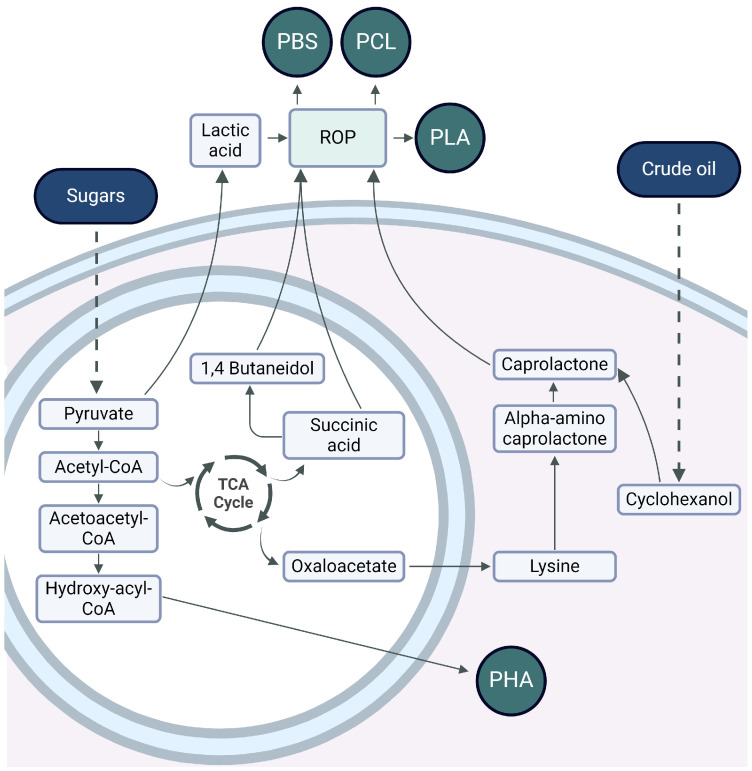
Metabolic pathways for the production of microbial polyesters. PHB: polybutylene succinate, PCL: poly(Ɛ-caprolactone), PLA: polylactic acid, PHA: polyhydroxyalkanoate.

PHA are biopolyesters synthesized intracellularly by microorganisms under excess carbon source and nutrient limitation conditions [39]. They are biodegradable, biocompatible, and can be synthesized from renewable carbon sources. Structurally, PHA monomers have a carboxyl group esterified to create bonds with the hydroxyl group of the next monomer and elongate the molecule to give rise to the polymer [40] (Table 1). Short chain length PHAs (scl-PHA) are thermoplastic polymers; their melting temperature is relatively high (180 °C), their transition temperature is between −5 °C and 20 °C, and their crystallinity is 60% to 80%. As for medium chain length PHAs (mcl-PHA), they are highly amorphous, with a transition temperature between −62 °C and −26 °C and a melting temperature between 42 °C and 58 °C, and are therefore classified as elastomers [41]. Numerous central pathways of microbial metabolism are involved in PHA synthesis, where acetyl-CoA plays a relevant role (Figure 2). The main route is based on the sugar degradation pathway from which acetyl-CoA is obtained. Through this pathway, the enzyme β-ketothiolase (PhaA) condenses two molecules of acetyl-CoA to form acetoacetyl-CoA. The NADPH-dependent enzyme acetoacetyl-CoA reductase (PhaB) acts on acetoacetyl-CoA to form (R)-3-hydroxybutyryl-CoA. Finally, PHA synthase (PhaC) catalyzes polymerization through esterification, producing PHA intracellularly in the cell [42].

#### 3.1.2. Polycaprolactone

PCL is an aliphatic, semi-crystalline polyester with a fairly low melting point of around 60 °C, and its hydrophobic character makes it soluble in organic solvents. It comprises a sequence of methylene units, between which ester groups are formed [43] (Table 1). PCL has been investigated for its mechanical properties, biodegradability, and miscibility with other polymers. It can be synthesized from ε-caprolactone by two main routes [44]. The first route starts with obtaining cyclohexanol from petroleum. The interaction of cyclohexanol with oxidizing agents, such as cyclohexanone monooxygenase or peracetic acid, produces cyclohexanone, a precursor of ε-caprolactone [45]. Finally, through ring-opening polymerization (ROP), the synthesis of PCL is achieved, which is based on the coordination-insertion mechanism proceeding to a metal catalyst and the formation of a growing metal-bound species [46]. The second route originates from the fermentation of sugars to obtain lysine, which will act as an intermediate for α-amino ε-caprolactone. Then, exposing this intermediate to high temperatures and pressures, ε-caprolactone will be obtained as a product, thus producing PCL via ROP [44] (Figure 2).

#### 3.1.3. Polybutylene Succinate

PBS is a bio-based aliphatic polyester obtained from the condensation polymerization of dicarboxylic acids [47] (Table 1). This biopolymer shows excellent biodegradability, thermoplastic processability between 45 °C and 10 °C and a melting temperature between 90 °C and 120 °C. To produce PBS, the polymerization of succinic acid (SA) and 1,4-butanediol (BD) is necessary, which are usually obtained from petroleum but also by bacterial fermentation. In recent years, several microorganisms have been studied and tested for producing SA by biotechnological processes with high yields. The SA obtained can be converted to BD by hydrogenation and then lead to esterification to produce PBS by ROP or polycondensation (Figure 2) [47]. ROP or polycondensation reactions occur in two stages: in the first stage, esterification reactions take place with water (or methanol) removal, while in the second stage, the temperature is raised, and the pressure is reduced to remove BD [47,48]. The synthesis is usually performed in a reactor, which is heated to 160–190 °C to initiate esterification under stirring, and when no more water (or alcohol) is distilled at normal pressure, the reaction is continued at high temperatures (220 °C). Due to the ability of SA to originate commodity products, such as BD, plants dedicated to the industrial-scale production of BD from sugars have been studied and installed using different bacteria [47].

#### 3.1.4. Polylactic Acid

PLA is an aliphatic polyester whose main structure consists of a carboxyl group and a methyl radical attached to the polymer chain’s methyl group [49] (Table 1). It is obtained from lactic acid produced from sugars. It has been among the most explored biopolymers due to its interesting properties, such as good tensile strength, resistance to bending, and biodegradability. PLA synthesis begins with sugar fermentation by bacteria. By this route, the microorganisms carry out glycolysis to obtain pyruvate as a precursor of lactic acid utilizing lactate dehydrogenase. Then, two very different methods can be used to obtain PLA from lactic acid: polycondensation or ROP [50]. The latter process requires several purification steps, which makes the method more expensive, but the final properties of the PLA obtained are of high molecular weight (Figure 2).

#### 3.1.5. Other Polyesters

Among the polyesters, there is a group with a high degree of biodegradability, but this has not yet been explored in packaging. This is the case of polyglycolic acid (PGA) and poly(lactic-co-glycolic acid) (PLGA), where the glycolic acid monomer of PGA is used as a co-monomer for the synthesis of PLGA to balance the mechanical strength of PLA [51].

Among the characteristics of PGA are its mechanical properties, biodegradability, biocompatibility, and high crystallinity; this is because it has a chemical structure like PLA but without the methyl group, which allows greater compactness in the chains [51]. It is mainly used in biomedicine, and its applications include resorbable sutures [52] and suture anchors for joint reconnection, allowing in situ absorption without requiring secondary surgical excision [53]. PLGA is a biodegradable and biocompatible polymer, is one of the most studied and is approved by the FDA (Food and Drug Administration). The main advantage of this polymer is its complete biodegradation in aqueous media. Its applications include the manufacture of sutures, drug delivery, and nanoparticles for controlled delivery [54].

**Table 1:** Most used commercial polyesters, types of packaging, and main antimicrobial agents.

Polyester	Chemical Structure	Type of Packaging	Antifungal Compound	References
PHA	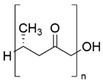	▪ Biofilms ▪ Wraps ▪ Disposable cups ▪ Shampoo bottles	▪ Silver nanoparticles (AgNPs) ▪ Eugenol (essential oil) ▪ Citrus peel extracts ▪ MgONPs	[55,56,57,58,59,60,61]
PCL	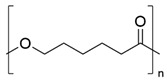	▪ Blown films ▪ Laminates and packaging ▪ Foamed packaging ▪ Wrapping for both direct ▪ and indirect food contact ▪ Biodegradable ▪ bags, films, and trays	▪ Grapefruit seed extract ▪ Thymol (derived from thyme essential oil) ▪ Zinc oxide (ZnO) ▪ Silver kaolinite ▪ Oregano essential oil ▪ Lavandula luisieri essential oil	[62,63,64,65]
PBS	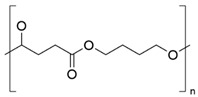	▪ Food packaging ▪ Disposable cutlery, households, bottles	▪ Bio-silver ▪ Zinc oxide (ZnO) ▪ Curcumin and carvacrol ▪ Carvacrol or cimophenol (oregano oil)	[66,67,68,69,70,71,72]
PLA	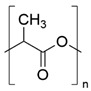	▪ Drinking cups, sundaes cups, and salad cups ▪ Overwrap and lamination films ▪ Blister packages ▪ Bottles, biofilms, bags, trays, ▪ cardboard cups	▪ Nisin ▪ Magnesium oxide (MgO) ▪ Zinc oxide (ZnO) ▪ Silver nanoparticles (AgNPs). ▪ CuO-TiO_2_ ▪ Cinnamaldehyde and carvacrol	[73,74,75,76,77]

### 3.2. Polysaccharides

Polysaccharides are carbohydrate polymers composed of many repeating monosaccharide units connected by glycosidic bonds and can acquire linear or branched structures [78]. They can be obtained from different sources, either from plants or industrial processes. These polymers are characterized as biodegradable and biocompatible, characteristics that, combined with their film-forming and gel-forming properties, have enabled their use to produce thin membrane films and gels for applications in food, medicine, and pharmaceuticals [79]. In Table 2, the most commonly used polysaccharides are shown.

#### 3.2.1. Cellulose

Cellulose is an abundant organic polymer. It is one of the main components of the cell membrane of plant tissues, together with lignin and hemicellulose [80]. It is a linear polysaccharide made up of glucose molecules via β-1,4-glycosidic linkages [81]. Although cellulose is one of the main components of the cell wall, it can also be synthesized by bacteria. Its strength and high purity characterize cellulose, so it does not require refining treatment. It is a biodegradable biopolymer, an ideal substitute for fossil fuel derivatives, which harm the environment [82].

Cellulose nanocrystals have been used for food packaging because they can generate compounds with excellent integrity and resistance when combined with other components. The use of mango crop residues to obtain nanocellulose and chitosan for producing a film with antifungal activity was studied [83]. The antifungal properties of the film were evaluated using mango in postharvest conditions with the fungi *Colletotrichum gloseosporioides* and *Lasiodiplodia theobramae*, where a 70% inhibition of *C. gloseosporioides* was obtained [83]. Another study evaluated an active packaging with carboxymethylcellulose-chitosan nanocomposite to which 0.5%, 1%, and 2% concentrations of ZnO nanoparticles were incorporated [84]. These nanocomposites were tested on slices of white bread to study their shelf life when inoculated with *Aspergillus niger* at 25 °C. All samples showed fungal growth after 15 days except the white bread with 2% ZnO incorporated in the nanocomposite [84].

#### 3.2.2. Starch

Starch is a natural polysaccharide of plants, and its most important sources include cereals, rhizomes, roots, and tubers. This polymer is composed of glucose monomers linked by glycosidic bonds [85]. Natural starch is usually found in the form of granules, which are spherical, oval, or irregular in shape, ranging in diameter from 0.1 µm to 200 µm. These granules are insoluble in cold water but are capable of absorbing water if heated excessively [86]. Starch is a renewable, biodegradable, low-cost, and easily modified polysaccharide, which makes it an attractive alternative as a precursor to produce food packaging products. Another property of starch is its ability to be converted into a thermoplastic material, from which starch derivatives or new products such as films, bags, or food packaging can be obtained [79].

Bioactive biocomposites have been developed from a starch-based thermoplastic polymer called Mater-Bi, including chitosan, tripolyphosphate, and submicroparticles containing ungeremine, which is an alkaloid active against the fungus *Penicilium roqueforti* [87]. Other researchers prepared thermoplastic starch plates containing cinnamon oil emulsion at different concentrations in the presence of a chia mucilage extract. The fungus *B. cinerea* was incubated on these plates, yielding up to 66% inhibition in mycelial growth after 10 days of incubation at 25 °C with the highest concentration of cinnamon oil [88].

#### 3.2.3. Chitosan

Chitin is the second most abundant polysaccharide on the planet, found in the form of macrofibrils in the exoskeletons of mollusks and crustaceans, as well as in fungi and the cuticles of some insects [89]. Chitosan is a linear copolymer comprising β-1,4-glucosamine and N-acetylglucosamine units linked by β-1,4-glycosidic bonds, soluble in acidic aqueous solutions, where the amino groups protonates are present [87]. Chitosan is extracted using chemical and biological methods [90].

Due to these compounds’ inherent antimicrobial properties, chitosan and its derivatives have been investigated for their uses in the biomedical, pharmaceutical, biotechnology, and food industries. At low pH, the amino groups of chitosan form a higher cationic charge density. This forms a high affinity towards negatively charged biological membranes, preventing normal cell metabolism and leading to cell death [91]. Due to their antimicrobial, biodegradable, non-toxic, biocompatible, and high adhesiveness properties, chitosan and its derivatives are used to develop active packaging, composite, and smart films.

Due to these remarkable properties, chitosan has been studied in various applications as an antimicrobial material. Nanocomposite chitosan films were developed utilizing chitosan extracted from shrimp shell residues and nanocellulose fibers obtained from agave bagasse. The films showed antibacterial activity by partially inhibiting the growth of *E.* coli, which was improved by adding silver nanoparticles to the films, achieving total growth inhibition of the bacteria [92]. Active chitosan films were produced using essential oil from basil, which was microencapsulated and grafted into chitosan edible films. Submerging slices of the films tested the antimicrobial effect of the films in *Staphylococcus saprophyticus* 3S and *E. coli* culture broths, showing a significant reduction in the cell viability of both bacterial strains [93]. Consequently, to investigate the potential use of these active films as food packaging, cooked ham slices were wrapped with the films and placed in Petri dishes to carry out microbiological analyses at different times, obtaining a similar trend for enterobacteria, lactic acid bacteria, and aerobic mesophilic bacteria, except for yeast population, which remained unaffected to the effects of the films [93].

The antimicrobial capacity in chitosan–starch biopolymer has also been studied with the incorporation of glycerol and ZnO, where glycerol acts as a plasticizer and ZnO as an amplifier, having an antimicrobial effect on Gram-positive and Gram-negative bacteria [94].

**Table 2:** Most commonly used commercial polysaccharide-based polymers, types of packaging, and main antimicrobial agents.

Polymer	Chemical Structure	Type of Packaging	Antifungal Compound	References
Cellulose	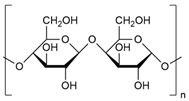	▪ Edible films ▪ Intelligent packaging ▪ Antifungal films ▪ Active packaging ▪ Edible coating	▪ Chitosan nanoparticles (CNP) ▪ Zinc oxide nanoparticles (ZnO NPs) ▪ Falcaria vulgaris extract ▪ Cavracol	[95,96,97,98,99,100,101,102]
Starch	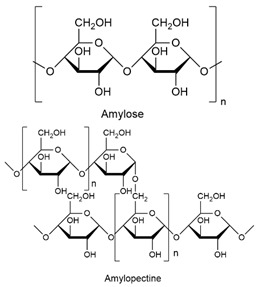	▪ Mixed films ▪ Antifungal films ▪ Multilayer films ▪ Intelligent films	▪ Trans-2-hexenal ▪ Chitosan tripolyphosphate sub-micro particles containing ungeremine (CTUn) ▪ Cinnamon oil stabilized by murcilage ▪ Rice husk ▪ Thymus vulgaris ▪ Tea polyphenols ▪ Zanthoxylum armatum essential oil	[95,103,104,105,106,107,108,109,110]
Chitosan	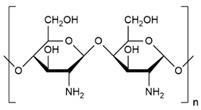	▪ Active packaging ▪ Antimicrobial packaging ▪ Edible films ▪ Smart films	▪ Chitosan nanoparticles (CNP) ▪ Zinz oxide nanoparticles (ZnO NPs) ▪ Grapefruit seed extract ▪ TiO_2_ ▪ Thymus vulgaris ▪ Titanium dioxide	[95,111,112,113,114,115,116,117,118,119,120]

#### 3.2.4. Polysaccharide-Based Aerogels

Aerogels are synthetic, porous, and ultralight materials obtained from a gel whose liquid content is replaced by a gas while maintaining its structure. They can be made from inorganic compounds, synthetic polymers, proteins, and polysaccharides (e.g., cellulose, starch, and chitosan) and can be applied as adsorbents for carbon dioxide, dyes, heavy metals, thermal insulators, and food packaging [121]. One of the areas where polysaccharide-based aerogels are most widely used is drug delivery, where these biopolymers act as drug-carrier matrices, providing an amorphous state that suppresses recrystallization. Another area of great importance is food, where polysaccharide-based aerogels can be used as dietary fiber, energy sources, or mechanical support structures in food packaging [122].

One of the most widely used drying techniques for producing aerogels is the supercritical CO_2_ (sc-CO_2_) drying process, where CO_2_ is used to transform the hydrogel solvent into a supercritical solution. This technique consists of three steps: gelatinization, aerogel formation, and drying. In the last step, the sc-CO2 is transferred to the liquid solvent in the gel, causing expansion and removal of excess liquid from the gel network. This is followed by depressurization at atmospheric pressure to recover the aerogel [121]. Another technique used for drying is freeze-drying. Freeze-drying is divided into pre-freezing, where the gel liquid can be frozen at −196 °C for 10 s or slowly frozen for 24 h at −18 °C, and then drying, where the frozen gel is dried by sublimation at −45 °C and 15 Pa for 48 h, generating a porous structure by the transition from liquid to gas [121].

Aerogels possess a porosity between 95% and 99.99%, a high specific surface area of more than 150 m^2^/g, and a density of 0.004 cm^3^ to 0.5 cm^3^ [121,122]. These characteristics allow it to be used as packaging material. Researchers have developed a carboxymethyl nanocellulose aerogel, supplemented with 75% glycerol and acrylamide, which also has antibacterial properties; by using a solution of chitosan and silver nanoparticles, a compressive strength of 90% was obtained, in addition to inhibition of microbial growth of *Escherichia coli* and *Staphylococcus aureus* [123]. Da Silva et al. developed and characterized physically cross-linked aerogels using germinated and non-germinated wheat starch, to which 6% *w*/*v* polyethylene oxide (PEO) was added. These aerogels showed degradation at approximately 300 °C and demonstrated a high adsorption capacity since after a 24-h immersion in water, the PEO-germinated wheat starch aerogel showed a mass increase of 1.14%, and the PEO-germinated non-germinated wheat starch aerogel showed a mass increase of 1.28% [124].

The development of biodegradable packaging materials has been strongly boosted in the last decades, finding two main groups: polyesters and polysaccharides. The selection of the type of biomaterial will depend on the complexity of the packaging and the type of application. There is an interest in the use of biomaterials in the form of blending to enhance the benefits regarding the synergism of such a mixture.

## 4. Synthetic Antifungal Compounds

Advances in the technology of biodegradable materials based on polyesters and the development of antimicrobial agents have led to the study of incorporating antifungal substances in food packaging. The antifungal mechanisms of action are varied depending on the agent used; however, the vast majority affect the cell membrane [125]. This section mentions three groups of synthetic compounds commonly applied as antifungals: metal nanoparticles, polyenes, and azoles. Their structure and main mode of action are explained and discussed.

### 4.1. Metal Nanoparticles

Materials classified as nanoparticles (NPs) usually have a size of less than 100 nm; however, materials on a scale above 500 nm have also received the same classification [126]. NPs have different specific physicochemical properties depending on their size scale, such as their optical properties; for example, metals platinum, gold, and silver have colors resembling red wine, yellowish gray, and black, respectively, when they are 20 nm in size [127]. Some materials, including nanorods, nanowires and nanofibers, have a diameter of less than 100 nm. Depending on how their aggregates are formed, they can have different ionic strength, valence, and optical properties [128], enabling different properties such as surface functionalization, accessible area, and porosity [129].

NPs and nanomaterials can be composed of metals, carbons, and polymers. The most studied NPs are Ag, Au, ZnO, and CuO, are known for their antimicrobial properties and, therefore, can be used in packaging. Depending on their antimicrobial properties, they can be divided into four main categories: antibacterial, antifungal, antiviral, and antiparasitic [130]. The functionalization of nanomaterial surfaces with antibiotics and inorganic disinfectants, such as metals, has gained popularity since the use of organic disinfectants with associated toxicity to humans is avoided [131].

Metallic NPs have been continuously questioned for their potential effects on human health, so their use still carries some concerns [132]. The toxicity of metal NPs is directly influenced by their type, exposure time, and sensitivity of the interaction zone [133]. They can induce toxicity through oxidative stress, inflammation, and physical disruption of cell membranes. Soluble components like metal ions can cause oxidative damage and enzyme inhibition. Among the most commonly used nanoparticles for packaging are Ag and Cu [134]. Ag NPs have been found to migrate into food containers in a range from 1.66 to 31.46 ng/cm^2^, which are below allowable limits [135]. Another study found that the total silver release could reach up to 3.1 ng/cm^2^ in acidic conditions, and total migration values remained below the permitted limit of 0.05 mg Ag/kg food according to EU Regulation [136]. Cu NPs dissolve and release copper ions, which can build up inside cells and, tear membranes and inactivate proteins. Copper nanoparticles have antibacterial qualities due to ion release [137,138]. No harmonized maximum limits of copper that can be added to food and food supplements are available in the EU regulation [139].

The European Commission’s (EC) statutory regulation EC 1935/2004, which states that the use of nanoparticles in packaged foods may not pose a risk to public health, is generally in charge of controlling their presence in food products throughout Europe (Article 3). Article 23 of Regulation EC 10/2011 states that NPs must be evaluated individually before being put on the market. A functional barrier must be used to meet a migration limit of 0.01 mg/kg if an unapproved chemical is used (Article 14, EC 450/2009) [140].

Among the mechanisms of action of the use of NPs are: (i) membrane damage through changes in the fungal cell wall, including surface shrinkage, cell aggregation, formation of pits and pores, and general deformation; (ii) intracellular damage through interaction with fungal DNA, including mitochondrial fragmentation, ribosome depolymerization, and chromatin damage. It is presumed that cell wall damage may cause DNA leakage outside the cell, but once NPs are inside the cell, some may intercalate with nucleic acids intracellularly; (iii) damage to hyphae and spores, (iv) inhibition in the process of biofilm formation; and (v) generation of ROS, mainly attributed to a decrease in membrane permeability [141]. ZnO NPs have been reported in PLA, decreasing the growth of microorganisms, including *E. coli*, *Salmonella typhi*, *S. aureus*, and *Candida albicans*, among others [142]. On the other hand, silver nanoplates arranged in PLA have been used to inhibit *E. coli* and *S. aureus* growth [143].

### 4.2. Polyenes

Polyenes are polyunsaturated organic compounds containing at least three C=C-type bonds, where the C=C bonds present conjugation, have unusual optical properties, and generate long hydrophobic segments [144]. The mechanism of action as an antifungal is to reduce the stability of the cell membrane by interacting with the hydrophobic rings of sterols ergosterol and cholesterol by Van der Waals forces. Examples of polyenes are nystatin and amphotericin. Ergosterol is present in fungal cells, while cholesterol is present in human cells, so it must be dosed discreetly in treating infections [145].

The use of nystatin and amphotericin B in the mcl-PHA bioplastic in the form of a film has been reported, presenting antimicrobial activity against filamentous fungi such as *Aspergillus fumigatus*, *Trochophyton mentagrophytes*, *Microsporum gypseum*, and *Candida* sp. [146]. Nystatin and amphotericin have been used in chitosan biopolymers, demonstrating growth inhibition of *C. albicans* and *Candida glabrata* [147].

### 4.3. Azoles

Azoles are a group of antifungal molecules that have a free imidazole ring in their structure, which is linked to other aromatic rings by a C-N type bond, depending on the number of nitrogen atoms, and can be divided into two groups: those with 3 N (e.g., fluconazole, itraconazole, and voriconazole) and 2 N in their rings (e.g., miconazole, ketoconazole, and clotrimazole) [148]. The mechanism of action is based on the inhibition of the enzyme 14-α-sterol demethylase, which allows the binding of ergosterol to the cell wall of the fungus [149,150]. These molecules have been reported to inhibit the growth of fungi, including *C. albicans*, decreasing their adhesion to polymers [151]. The use of fluconazole in chitosan has also been demonstrated in the biomedical field for transporting drugs into the body to be metabolized [152].

Antifungal compounds of synthetic origin are highly effective, with metal nanoparticles being the most widely used in the packaging industry. The mechanism of action is mainly focused on affecting the fungal cell wall, although it can also act by interacting with its DNA. The use of these antifungals may be questionable due to their potential toxicity effect on human health.

## 5. Natural Antifungal Compounds

Given the growing interest in bioactive compounds with antimicrobial properties, different metabolites contained in natural extracts have been explored and characterized. These bioactives are used as substitutes for chemical or synthetic additives to preserve the organoleptic properties of fruits. Within this group of natural compounds, we find the family of terpenes, essential oils, and various aromatic compounds, which are obtained from different parts of the plant, such as leaves, stems, roots, seeds, flowers, and fruits, using different extraction methods [153,154,155,156]. Different mechanisms can extract bioactive compounds. Among the most common processes are Soxhlet extraction, maceration, and hydrodistillation. At the same time, other less conventional but very effective methodologies are ultrasound-assisted extraction, enzymatic extraction, pulsed-electric field extraction, ultrasound, pressurized liquid extraction, supercritical fluid extraction, and supercritical fluid extraction [157].

Plants produce a variety of chemical compounds that can be divided into primary (core) and secondary metabolites. The latter is considered necessary for the survival of the plant since their fundamental function is plant-environment interaction and adaptation (e.g., edaphoclimatic conditions), protection against pathogens and insects or from other biotic or abiotic stresses, as well as the attraction of pollinators and frugivores, allelopathy, and signaling [158,159,160,161]. In plants, it is known that secondary metabolites comprise less than 1% of the total carbon in plant molecules, which have been divided into three main groups of nitrogen or sulfur-containing molecules, including phenolic compounds, terpenoids/isoprenoids, and alkaloids or glucosinolates. Most of these metabolites are produced from glycolysis, the tricarboxylic acid cycle (TCA), aliphatic amino acids, the pentose phosphate pathway, the shikimate pathway, and notably aromatic amino acids (AAAs) [158].

In plants, phenolic compounds are the largest class of secondary metabolites, and they are synthesized from aromatic amino acids, such as phenylalanine by phenylpropanoid. They have various structures found in free form and conjugation with sugar moieties [162]. These are further subdivided into phenolic acids, flavonoids (e.g., flavones, isoflavones, flavanones, and flavonols), anthocyanidins, and tannins. These secondary metabolites are extremely diverse in terms of structure and biosynthetic pathways, including more than 20,000 distributed through different molecules in approximately 20% of known vascular plants [163,164,165]. They act as defense molecules and protect plants from pathogens and herbivores.

### 5.1. Terpenes

Terpenes, also called isoprenoids, are the second most numerous compounds after polyphenols, with at least 35,000 being characterized to date. Terpenes are hydrocarbons synthesized from isoprene subunits by condensation and cyclization reactions, and their classification depends on the number of isoprene units in their carbon structure [166]. Among the best-known terpenes are geraniol and limonene (monoterpenes), humulene and farnesol (sesquiterpenes), and cembrene and taxadiene (diterpenes) [167].

One of the best-known components is carvacrol, which has a high activity due to the presence of a hydroxyl group in the ortho position to its methyl group, allowing delocalization of the electron (resonance). This contrasts with thymol, which has a hydroxyl group in the meta position. Carvacrol and thymol can be obtained from *Origanum dictamnus* oils [168], an essential oil further discussed in Section 5.3. The mechanism of action of carvacrol as an antifungal is based on the interruption of ion homeostasis in yeast, affecting the concentration of Ca^2+^ [169]. Another antifungal activity of carvacrol has been studied in *C. albicans*. Carvacrol disrupts endoplasmic reticulum integrity, disturbing the cell membrane biology, such as permeability and lipid content [170].

Alpha-pinene is a bicyclic monoterpene found in eucalyptus and rosemary essential oils [171]. Antifungal activity of alpha-pinene associated with boric acid has been reported for *Candida* spp. isolated from patients with otomycosis [172]. Theoretical studies have also been carried out that suggest that it acts on the cell wall of *C. albicans*, interfering with the enzymes delta(14)-sterol reductase and 1,3-β-glucan synthase, preventing the formation of biofilm [173].

P-cymene (1-methyl-4-(1-methylethyl)-benzene) is a terpene of the alkylbenzene type with a short half-life [174,175]. Inhibition of the growth of *A. flavus* has been reported in conjunction with other terpenes, such as carvacrol and thymol [176]. p-Cymene and carvacrol have been shown to increase membrane size in *B. cereus* in the form of liposome aggregates, with p-cymene increasing membrane size 2.7 times more than carvacrol [177]. It has also been shown that p-cymene vapors have an antifungal effect on *Penicillium digitatum*, which presents synergism in the presence of the compound γ-terpinene [178].

Terpinen-4-ol and α-terpineol, the main components of tea tree oil (TTO), have shown important antifungal activity on *B. cinerea* by inhibiting mycelial growth and disrupting hyphae morphology. Their effectiveness is enhanced when used synergistically at a 1:1 ratio [179].

### 5.2. Propolis

Propolis is highly dependent on the region in which it is produced since it is influenced by variables, including plant material, climate, and geography of the sector [180], and it is widely known for its antimicrobial and antifungal activity [181]. In propolis, many molecules have antifungal properties, such as phenolic acids (e.g., ferulic, gallic, and vanillinic acids). Gallic acid is a trihydroxybenzoic acid that has been shown to inhibit the growth of *Fusarium graminearum* and *Candida* strains, where its mechanism of action was based on disrupting the cell membrane [182]. Ferulic acid corresponds to a hydroxycinnamic acid with antioxidant properties, in which antifungal activity has been reported through its amide derivatives on *C. albicans*, acting at the cell wall level [183]; meanwhile, antimicrobial activity on *Listeria monocytogenes* [184] and antibiotic enhancer activity against *Acinetobacter baumannii* were also reported [185]. Vanillinic acid is a monohydroxybenzoic acid derived primarily from plants, such as vanilla, garden cress, and paprika [186]. By measuring variations in intracellular ATP concentration, intracellular pH, membrane potential, and cell morphology it has been possible to elucidate the antimicrobial activity of vanillic acid against carbapenem-resistant *Enterobacter hormaechei* (CREH) [187].

### 5.3. Essential Oils

Essential oil (EO) is a mixture of aromatic, nonfatty compounds with apolar and polar characteristics and a molecular mass of less than 300 g/mol. The main categories of compounds are terpenes and terpenoids. The richness of EOs lies in the large number of chemical compounds that comprise them, their persistence, and their synergistic interaction ability. This makes their action on microorganisms more effective than each of their compounds separately [188]. The components of essential oils can be divided into four types: (1) according to its primary biosynthetic origin, (2) the number of carbons and size of the molecule, (3) the skeleton of the molecule, and (4) characteristics regarding the oxidation of electronegative atoms [189]. One of its main characteristics is its antimicrobial activity, which is compatible with packaging polymers to improve the preservation of the transported fruits.

EOs have been reported from different plants, including *Thymus vulgaris* (thyme), *Origanum vulgare* (oregano), and *Origanum dictamus* (dictamus), and *Melaleuca alternifolia* (tea tree), among others. The most effective EOs used in antifungal applications are summarized in Table 3). The antifungal effect of EOs has been attributed to the presence of phenolic compounds, such as pinene, myrcene, carene, and terpinene [190], and the wide family of terpenes previously discussed in Section 5.1.

Among the EOs studied to control the growth of *B. cinerea, Origanum heracleoticum* and *Thymus vulgaris* demonstrated high antifungal activity, causing cell structure alteration, hyphal morphology disruption, and cell apoptosis [191]. Another study tested 26 EOs for *Rhizopus* rot on strawberry and peach fruits, concluding that three, including *Mentha piperita*, *Mentha spicata*, and *Thymus vulgaris*, exhibited significant results. They triggered cell membrane disruption through plasma membrane disturbance, hyphal morphology alteration, and endogenous ROS production stimulation in *R. stolonifera* [192]. Jiayu Xu et al. conducted a metabolomic analysis of *B. cinerea* treated with TTO, demonstrating that TTO disrupts the TCA and alters the regulation of the membrane and cellular components, thus causing cell membrane leakage, interfering with mitochondrial function and oxidative stress [193].

**Table 3:** Essential oils as natural antifungal agents.

Essential oils	Application Method	Mechanism of Action	Target Microorganism	References
*Melaleuca alternifolia*	▪ Contact, concentration at 5 mL/L. ▪ Contact, concentrations at 0.25, 0.5, 1.0, and 1.5 μL/mL. ▪ Volatile and contact, concentrations at 0.1, 0.4, 0.7, and 1.0 mL/L.	▪ Tricarboxylic acid cycle disruption. ▪ Cell membrane disruption. ▪ Mitochondrial dysfunction. ▪ Oxidative stress.	▪ *Botrytis cinerea*	[179,193,194]
*Mentha piperita, Mentha spicata*	▪ Volatile, concentration at 150 μL/L.	▪ Cell membrane disruption. ▪ Hyphal morphology alteration. ▪ Production of reactive oxygen species activation	▪ *Rhizopus stolonifer* ▪ *Botrytis cinerea*	[192,195]
*Thymus vulgaris*	▪ Volatile, concentration at 150 μL/L.	▪ Cell membrane disruption. ▪ Hyphal morphology alteration. ▪ Production of reactive oxygen species activation	▪ *Rhizopus stolonifer* ▪ *Botrytis cinerea*	[192,196,197,198]
*Origanum heracloeticum, Origanum vulgare*	▪ Volatile, concentrations at 150, 100, and 50 μL/L.	▪ Cell structure alteration. ▪ Hyphal morphology disruption. ▪ Cell apoptosis	▪ *Botrytis cinerea*	[191,197,198]
*Citrus sinensis*	▪ Contact, concentrations of 0.1, 0.3, 0.5, 0.7, 1.0, 1.5, 2.0, 2.5, 3.0, and 3.5 μg oil/mL.	▪ Hyphal morphology disruption. ▪ Cell membrane disruption	▪ *Aspergillus niger*	[199]

Due to their volatile feature, EOs and their compounds offer an important alternative for fungal control in food packaging. That creates the possibility of a slow vapor release during storage and transportation, exerting their antifungal properties more effectively and preserving food flavors, fruit firmness, and preventing fruit weight loss.

Antifungal compounds of natural origin have been widely used in packaging applications due to their biocompatibility. In addition, the effectiveness of different natural compounds, such as extracts of organic origin and essential oils, is highlighted, where the synergistic effect of a set of volatile compounds is often highly inhibitory to fungal growth.

## 6. Bibliometric Analysis of Bio-Based Packaging with Antifungal Properties

Due to environmental concerns and regulations in the packaging industry worldwide, scientific studies have been focused on bio-based packaging with antifungal properties. An alternative for exploring this scientific trend is through a bibliometric analysis approach, a well-established statistical tool widely used for mapping many scientific studies and providing an integrated overview of a research field.

Here, a bibliometric analysis of bio-based packaging with antifungal properties was conducted to analyze the scientific evolution of this topic between 2000 and 10 May 2024.

### 6.1. Database Selection, Search Strategy, and Data Extraction

The data for the bibliometric analysis was obtained from the Scopus database. The search equation was used in the “article title, abstract, and keywords” and further refined to article and review as the document type. To address most of the articles related to bio-based packaging with antifungal properties, the terms “bio-packaging” and “biodegradable” were joined by the Boolean operator “OR” and further combined with “antifungal” by using the Boolean operator “AND”. No restrictions in terms of language were applied.

The search equation designed had the following syntax:

(TITLE-ABS-KEY (bio-packaging) OR TITLE-ABS-KEY (biodegradable) AND TITLE-ABS-KEY (antifungal)) AND PUBYEAR > 1999 AND PUBYEAR < 2025 AND (LIMIT-TO (DOCTYPE, “ar”) OR LIMIT-TO (DOCTYPE, “re”)).

The information retrieved from Scopus during the search were (1) citation and bibliographical information, and (2) abstract and keywords. All were exported to Microsoft Excel^®^, version 2110. Later, VOSviewer 1.6.20 was used for visualization and data analysis.

### 6.2. Global Scientific Output Related to Bio-Based Packaging with Antifungal Properties

**Figure 3:**
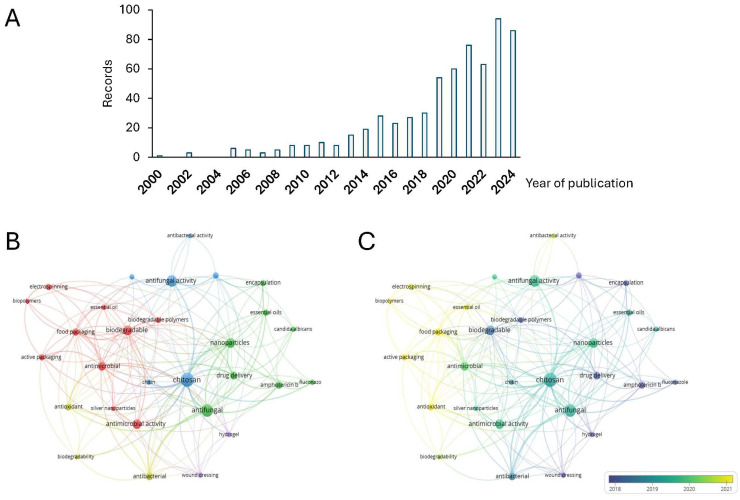
Bibliometric network of studies related to bio-based packaging with antifungal properties between 2000 and 15 September 2024. (**A**) Evolution in the number of records between 2000 and 15 September 2024. (**B**) Research topic map. (**C**) Research topic map with time overlap. Note: the minimum number of occurrences of a keyword was set to 10.

The search process yielded 632 records, of which 81.3% and 18.7% were research articles and reviews, respectively. Figure 3A displays the evolution in the number of records between 2000 and 15 September 2024. There has been an increase in the worldwide production of scientific publications focused on this topic, which has accelerated in the last decade (mainly since 2010). The main areas of knowledge in which the issued papers were classified were as follows: (1) biochemistry, genetics, and molecular biology; (2) materials science; (3) chemistry; (4) pharmacology, toxicology, and pharmaceutics; and (5) agricultural and biological sciences with 14.6%, 13.3%, 13.1%, 10.6%, and 9.7% of the records, respectively. India, China, and the United States were the leading countries regarding the number of publications. The top five were rounded out by Brazil and Poland with 145, 61, 59, 44, and 42 records, respectively.

### 6.3. Bibliometric Networks of Bio-Based Packaging with Antifungal Properties

Figure 3B shows the analysis of the co-occurrence of the most used author’s keywords among the papers published in the period evaluated. The bibliometric network contains 29 nodes within 5 clusters (color-differentiated). In this regard, consider the following notes to interpret Figure 3.

- The nodes represent the author’s keywords.
- The node’s size indicates their occurrences, which are the number of papers with the corresponding term in their title or abstract.
- The width of the line linking the nodes is proportional to the strength of the relationship between the author’s keywords.
- The distance between nodes indicates the relatedness of them.

The red and blue groups can be labeled as food packaging, essential oils, biodegradable polymers and films, and antimicrobial and antifungal activities. In addition, the most active topics in this area are, in fact, food and active packaging, and essential oils. Thus, these nodes could be considered an emerging theme in this field.

Although the term chitosan does not appear in the designed search equation, this node is one of the largest in the bibliometric network, indicating the relevance of this biopolymer in different research areas, not only in the food industries.

EOs are an emerging topic that is positioned in one of the extremes of the network. When reviewing this node in Figure 3C in the temporal overlap map, it is in yellow, which means that the studies have been concentrated in the last five years. Also, the food packaging and biopolymer labels are in yellow.

## 7. Conclusions and Perspectives

Preserving fruits and vegetables with biomaterials is the way of the future to guarantee their quality and provide a sustainable solution with a human health safety seal. In the last decades, different sustainable antifungal packaging alternatives have been developed, including elaborating materials based on biodegradable polymers with antimicrobial activity and/or adding biomolecules or bio-based compounds. Both synthetic and natural antifungal compounds have been evaluated on a variety of bio-based materials; however, the great effectiveness of natural antifungals is highlighted, thus encouraging the use of compounds that contribute to the circularity of the packaging industry. The main challenges for synthetic compounds lie in the potentially harmful effects on health and the environment, while for natural compounds, it is usually the applicability on an industrial scale and is able to maintain the antifungal effects over time. In this sense, the industry faces important challenges in terms of sustainable development when incorporating biopolymers into production lines. Although there are complex packaging materials, such as multilayer packaging, where biopolymers do not yet meet the high technical requirements demanded, there are multiple applications where replacing bio-based or biodegradable matrices is feasible.

## References

[1] Jambeck J.R., Geyer R., Wilcox C., Siegler T.R., Perryman M., Andrady A., Narayan R., Law K.L. (2015). Plastic waste inputs from land into the ocean. Science.

[2] Jacobsen L.F., Pedersen S., Thøgersen J. (2022). Drivers of and barriers to consumers’ plastic packaging waste avoidance and recycling—A systematic literature review. Waste Manag..

[3] Sundqvist-Andberg H., Åkerman M. (2021). Sustainability governance and contested plastic food packaging—An integrative review. J. Clean. Prod..

[4] Marsh K., Bugusu B. (2007). Food packaging—Roles, materials, and environmental issues: Scientific status summary. J. Food Sci..

[5] Moshood T.D., Nawanir G., Mahmud F., Mohamad F., Ahmad M.H., AbdulGhani A. (2022). Biodegradable plastic applications towards sustainability: A recent innovations in the green product. Clean. Eng. Technol..

[6] Cruz R.M.S., Krauter V., Krauter S., Agriopoulou S., Weinrich R., Herbes C., Scholten P.B.V., Uysal-Unalan I., Sogut E., Kopacic S. (2022). Bioplastics for Food Packaging: Environmental Impact, Trends and Regulatory Aspects. Foods.

[7] Bandyoypadhyay S., Saha N., Brodnjak U.V., Saha P. (2018). Bacterial cellulose based greener packaging material: A bioadhesive polymeric film. Mater. Res. Express.

[8] Gu J.-D. (2021). Biodegradability of plastics: The issues, recent advances, and future perspectives. Environ. Sci. Pollut. Res..

[9] Guajardo C., Andler R. (2024). Challenges and perspectives in enzymatic polymer fragmentation: The case of rubber and polyethylene terephthalate. J. Clean. Prod..

[10] Hussein Z., Fawole O.A., Opara U.L. (2020). Harvest and Postharvest Factors Affecting Bruise Damage of Fresh Fruits. Hortic. Plant J..

[11] Flores-López M.L., Cerqueira M.A., de Rodríguez D.J., Vicente A.A. (2016). Perspectives on Utilization of Edible Coatings and Nano-laminate Coatings for Extension of Postharvest Storage of Fruits and Vegetables. Food Eng. Rev..

[12] Luesuwan S., Naradisorn M., Shiekh K.A., Rachtanapun P., Tongdeesoontorn W. (2021). Effect of active packaging material fortified with clove essential oil on fungal growth and post-harvest quality changes in table grape during cold storage. Polymers.

[13] Fang Y., Wakisaka M. (2021). A Review on the modified atmosphere preservation of fruits and vegetables with cutting-edge technologies. Agriculture.

[14] Onwude D.I., Chen G., Eke-Emezie N., Kabutey A., Khaled A.Y., Sturm B. (2020). Recent advances in reducing food losses in the supply chain of fresh agricultural produce. Processes.

[15] Seyedmousavi S. (2019). Aspergillosis in humans and animals. Recent Trends in Human and Animal Mycology.

[16] Sridhar A., Ponnuchamy M., Kumar P.S., Kapoor A. (2021). Food preservation techniques and nanotechnology for increased shelf life of fruits, vegetables, beverages and spices: A review. Environ. Chem. Lett..

[17] Nian L., Wang M., Sun X., Zeng Y., Xie Y., Cheng S., Cao C. (2022). Biodegradable active packaging: Components, preparation, and applications in the preservation of postharvest perishable fruits and vegetables. Crit. Rev. Food Sci. Nutr..

[18] Dai L., Wang X., Mao X., He L., Li C., Zhang J., Chen Y. (2024). Recent advances in starch-based coatings for the postharvest preservation of fruits and vegetables. Carbohydr. Polym..

[19] Maurizzi E., Bigi F., Quartieri A., De Leo R., Volpelli L.A., Pulvirenti A. (2022). The Green Era of Food Packaging: General Considerations and New Trends. Polymers.

[20] Kahramanoğlu İ., Chen C., Rengasamy K.R., Wan C. (2020). The safety future of fruit preservation with biomaterials. Hortic. Int. J..

[21] Pergomet J.L., Di Liberto M.G., Derita M.G., Bracca A.B., Kaufman T.S. (2018). Activity of the pterophyllins 2 and 4 against postharvest fruit pathogenic fungi. Comparison with a synthetic analog and related intermediates. Fitoterapia.

[22] Bano A., Gupta A., Prusty M.R., Kumar M. (2023). Elicitation of Fruit Fungi Infection and Its Protective Response to Improve the Postharvest Quality of Fruits. Stresses.

[23] Filtenborg O., Frisvad J., Thrane U. (1996). Moulds in food spoilage. Int. J. Food Microbiol..

[24] You J., Zhang J., Wu M., Yang L., Chen W., Li G. (2016). Multiple criteria-based screening of Trichoderma isolates for biological control of Botrytis cinerea on tomato. Biol. Control.

[25] Seta S., Gonzalez M., Lori G. (2004). First report of walnut canker caused by *Fusarium incarnatum* in Argentina. Plant Pathol..

[26] Salem E.A., Youssef K., Sanzani S.M. (2016). Evaluation of alternative means to control postharvest Rhizopus rot of peaches. Sci. Hortic..

[27] Neri F., Donati I., Veronesi F., Mazzoni D., Mari M. (2010). Evaluation of *Penicillium expansum* isolates for aggressiveness, growth and patulin accumulation in usual and less common fruit hosts. Int. J. Food Microbiol..

[28] Prusky D., Alkan N., Mengiste T., Fluhr R. (2013). Quiescent and necrotrophic lifestyle choice during postharvest disease development. Annu. Rev. Phytopathol..

[29] Li D., Zhang X., Gu X., Zhang Q., Zhao L., Zheng X., Zhang H. (2019). The infection of grapes by *Talaromyces rugulosus* O1 and the role of cell wall-degrading enzymes and ochratoxin A in the infection. Physiol. Mol. Plant Pathol..

[30] Soyer J.L., Hamiot A., Ollivier B., Balesdent M., Rouxel T., Fudal I. (2015). The APSES transcription factor LmStuA is required for sporulation, pathogenic development and effector gene expression in *Leptosphaeria maculans*. Mol. Plant Pathol..

[31] Thynne E., Saur I.M.L., Simbaqueba J., Ogilvie H.A., Gonzalez-Cendales Y., Mead O., Taranto A., Catanzariti A., McDonald M.C., Schwessinger B. (2017). *Fungal phytopathogens* encode functional homologues of plant rapid alkalinization factor (RALF) peptides. Mol. Plant Pathol..

[32] Sung S.-Y., Sin L.T., Tee T.-T., Bee S.-T., Rahmat A., Rahman W., Tan A.-C., Vikhraman M. (2013). Antimicrobial agents for food packaging applications. Trends Food Sci. Technol..

[33] Suzuki M., Tachibana Y., Kasuya K.-I. (2021). Biodegradability of poly(3-hydroxyalkanoate) and poly(ε-caprolactone) via biological carbon cycles in marine environments. Polym. J..

[34] Bading M., Olsson O., Kümmerer K. (2024). Analysis of environmental biodegradability of cellulose-based pharmaceutical excipients in aqueous media. Chemosphere.

[35] Gamage A., Thiviya P., Mani S., Ponnusamy P.G., Manamperi A., Evon P., Merah O., Madhujith T. (2022). Environmental Properties and Applications of Biodegradable Starch-Based Nanocomposites. Polymers.

[36] Rasak A., Heryanto H., Tahir D. (2024). High degradation bioplastics chitosan-based from scale waste of milkfish (*Chanos chanos*). Int. J. Biol. Macromol..

[37] Aliotta L., Seggiani M., Lazzeri A., Giganate V., Cinelli P. (2022). A brief review of Poly (Butylene Succinate) (PBS) and Its Main Copolymers: Synthesis, Blends, Composites, Biodegradability, and Applications. Polymers.

[38] Ibrahim N.I., Shahar F.S., Sultan M.T.H., Shah A.U.M., Safri S.N.A., Yazik M.H.M. (2021). Overview of Bioplastic Introduction and Its Applications in Product Packaging. Coatings.

[39] Berezina N., Yada B. (2016). Improvement of the poly(3-hydroxybutyrate-co-3-hydroxyvalerate) (PHBV) production by dual feeding with levulinic acid and sodium propionate in *Cupriavidus necator*. New Biotechnol..

[40] Pereira J.R., Araújo D., Freitas P., Marques A.C., Alves V.D., Sevrin C., Grandfils C., Fortunato E., Reis M.A., Freitas F. (2021). Production of medium-chain-length polyhydroxyalkanoates by *Pseudomonas chlororaphis* subsp. *aurantiaca*: Cultivation on fruit pulp waste and polymer characterization. Int. J. Biol. Macromol..

[41] Koller M. (2017). Advances in polyhydroxyalkanoate (PHA) production. Bioengineering.

[42] Anjum A., Zuber M., Zia K.M., Noreen A., Anjum M.N., Tabasum S. (2016). Microbial production of polyhydroxyalkanoates (PHAs) and its copolymers: A review of recent advancements. Int. J. Biol. Macromol..

[43] Mochane M.J., Magagula S.I., Sefadi J.S., Mokhena T.C. (2021). A review on green composites based on natural fiber-reinforced polybutylene succinate (PBS). Polymers.

[44] Beerthuis R., Rothenberg G., Shiju N.R. (2015). Catalytic routes towards acrylic acid, adipic acid and ε-caprolactam starting from biorenewables. Green Chem..

[45] Bińczak J., Szelwicka A., Siewniak A., Dziuba K., Chrobok A. (2022). Oxidation of Cyclohexanone with Peracids—A Straight Path to the Synthesis of ε-Caprolactone Oligomers. Materials.

[46] Guarino V., Gentile G., Sorrentino L., Ambrosio L. (2017). Polycaprolactone: Synthesis, Properties, and Applications. Encyclopedia of Polymer Science and Technology.

[47] Labruyère C., Talon O., Berezina N., Khousakoun E., Jérôme C. (2014). Synthesis of poly(butylene succinate) through oligomerization-cyclization- ROP route. RSC Adv..

[48] Gigli M., Fabbri M., Lotti N., Gamberini R., Rimini B., Munari A. (2016). Poly(butylene succinate)-based polyesters for biomedical applications: A review. Eur. Polym. J..

[49] Ren J. (2010). Biodegradable Poly(Lactic Acid): Synthesis, Modification, Processing and Applications.

[50] Rawoof S.A.A., Kumar P.S., Vo D.-V.N., Devaraj K., Mani Y., Devaraj T., Subramanian S. (2021). Production of optically pure lactic acid by microbial fermentation: A review. Environ. Chem. Lett..

[51] Samantaray P.K., Little A., Haddleton D.M., McNally T., Tan B., Sun Z., Huang W., Ji Y., Wan C. (2020). Poly(glycolic acid) (PGA): A versatile building block expanding high performance and sustainable bioplastic applications. Green Chem..

[52] Colwell J., Halley P., Varley R., Heidarian P., McNally T., Peijs T., Vandi L. (2024). Self-reinforced biodegradable thermoplastic composites. Adv. Compos. Hybrid Mater..

[53] Wang J.-J., Zhou Y.-G., Zhang Q.-Q., Zou J. (2024). Synthesis and properties of novel degradable polyglycolide-based polyurethanes. e-Polymers.

[54] Mir M., Ahmed N., Rehman A.U. (2017). Recent applications of PLGA based nanostructures in drug delivery. Colloids Surf. B Biointerfaces.

[55] Castro-Mayorga J.L., Martínez-Abad A., Fabra M.F., Lagarón J.M., Ocio M.J., Sánchez G. (2016). Silver-Based Antibacterial and Virucide Biopolymers: Usage and Potential in Antimicrobial Packaging. Antimicrobial Food Packaging.

[56] Figueroa-Lopez K.J., Cabedo L., Lagaron J.M., Torres-Giner S. (2020). Development of Electrospun Poly(3-hydroxybutyrate-*co*-3-hydroxyvalerate) Monolayers Containing Eugenol and Their Application in Multilayer Antimicrobial Food Packaging. Front. Nutr..

[57] Garrido-Miranda K.A., Rivas B.L., -Rivera M.A.P., Sanfuentes E.A., Peña-Farfal C. (2018). Antioxidant and antifungal effects of eugenol incorporated in bionanocomposites of poly(3-hydroxybutyrate)-thermoplastic starch. LWT.

[58] Kuntzler S.G., de Almeida A.C.A., Costa J.A.V., de Morais M.G. (2018). Polyhydroxybutyrate and phenolic compounds microalgae electrospun nanofibers: A novel nanomaterial with antibacterial activity. Int. J. Biol. Macromol..

[59] Kumari S.V.G., Pakshirajan K., Pugazhenthi G. (2024). Development and characterization of active poly (3-hydroxybutyrate) based composites with grapeseed oil and MgO nanoparticles for shelf-life extension of white button mushrooms (*Agaricus bisporus*). Int. J. Biol. Macromol..

[60] Rusková M., Šišková A.O., Mosnáčková K., Gago C., Guerreiro A., Bučková M., Puškárová A., Pangallo D., Antunes M.D. (2023). Biodegradable Active Packaging Enriched with Essential Oils for Enhancing the Shelf Life of Strawberries. Antioxidants.

[61] Fiorentini C., Garrido G.D., Bassani A., Cortimiglia C., Zaccone M., Montalbano L., Martinez-Nogues V., Cocconcelli P.S., Spigno G. (2022). Citrus peel extracts for industrial-scale production of bio-based active food packaging. Foods.

[62] Thakur M., Thakur M., Majid I., Majid I., Hussain S., Hussain S., Nanda V., Nanda V. (2021). Poly(ε-caprolactone): A potential polymer for biodegradable food packaging applications. Packag. Technol. Sci..

[63] Nasir N.N., Othman S.A. (2020). Application of Bioplastic Packaging in Industry. J. Adv. Res. Mater. Sci..

[64] Ferreira E.F., Mouro C., Silva L., Gouveia I.C. (2022). Sustainable Packaging Material Based on PCL Nanofibers and *Lavandula luisieri* Essential Oil, to Preserve Museological Textiles. Polymers.

[65] Shi C., Zhou A., Fang D., Lu T., Wang J., Song Y., Lyu L., Wu W., Huang C., Li W. (2022). Oregano essential oil/β-cyclodextrin inclusion compound polylactic acid/polycaprolactone electrospun nanofibers for active food packaging. Chem. Eng. J..

[66] Dicker M.P., Duckworth P.F., Baker A.B., Francois G., Hazzard M.K., Weaver P.M. (2014). Green composites: A review of material attributes and complementary applications. Compos. Part A Appl. Sci. Manuf..

[67] Wiburanawong S., Petchwattana N., Covavisaruch S. (2014). Carvacrol as an antimicrobial agent for poly(butylene succinate): Tensile properties and antimicrobial activity observations. Adv. Mater. Res..

[68] Aziman N., Kian L.K., Jawaid M., Sanny M., Alamery S. (2021). Morphological, structural, thermal, permeability, and antimicrobial activity of PBS and PBS/TPS films incorporated with biomaster-silver for food packaging application. Polymers.

[69] Petchwattana N., Covavisaruch S., Wibooranawong S., Naknaen P. (2016). Antimicrobial food packaging prepared from poly(butylene succinate) and zinc oxide. Measurement.

[70] Mohamad N., Mazlan M.M., Tawakkal I.S.M.A., Talib R.A., Kian L.K., Jawaid M. (2022). Characterization of Active Polybutylene Succinate Films Filled Essential Oils for Food Packaging Application. J. Polym. Environ..

[71] Łopusiewicz Ł., Macieja S., Bartkowiak A., El Fray M. (2021). Antimicrobial, antibiofilm, and antioxidant activity of functional poly(Butylene succinate) films modified with curcumin and carvacrol. Materials.

[72] Promhuad K., Phothisarattana D., Laorenza Y., Bumbudsanpharoke N., Harnkarnsujarit N. (2023). Zinc oxide enhanced the antibacterial efficacy of biodegradable PBAT/PBS nanocomposite films: Morphology and food packaging properties. Food Biosci..

[73] Ahmed J., Varshney S.K. (2011). Polylactides—Chemistry, properties and green packaging technology: A review. Int. J. Food Prop..

[74] Jin T., Zhang H. (2008). Biodegradable polylactic acid polymer with nisin for use in antimicrobial food packaging. J. Food Sci..

[75] Khalil A.M., El-Sayed S.M., Youssef A.M. (2023). Valorization of polylactic acid bionanocomposites enriched with CuO-TiO_2_ for packaging applications. Biomass-Convers. Biorefin..

[76] Gonon H., Srisa A., Promhuad K., Chonhenchob V., Bumbudsanpharoke N., Jarupan L., Harnkarnsujarit N. (2023). PLA thermoformed trays incorporated with cinnamaldehyde and carvacrol as active biodegradable bakery packaging. Food Packag. Shelf Life.

[77] Oyama I.M.C., dos Anjos E.G.R., Morgado G.F.d.M., Martins E.F., Passador F.R. (2023). Antistatic and antimicrobial packaging of PLA/PHBV blend-based graphene nanoplatelets nanocomposites. J. Appl. Polym. Sci..

[78] El-Naggar M.E., Othman S.I., Allam A.A., Morsy O.M. (2020). Synthesis, drying process and medical application of polysaccharide-based aerogels. Int. J. Biol. Macromol..

[79] Nešić A., Cabrera-Barjas G., Dimitrijević-Branković S., Davidović S., Radovanović N., Delattre C. (2020). Prospect of Polysaccharide-Based Materials as Advanced Food Packaging. Molecules.

[80] Mishra S., Singh P.K., Pattnaik R., Kumar S., Ojha S.K., Srichandan H., Parhi P.K., Jyothi R.K., Sarangi P.K. (2022). Biochemistry, Synthesis, and Applications of Bacterial Cellulose: A Review. Front. Bioeng. Biotechnol..

[81] McNamara J.T., Morgan J.L.W., Zimmer J. (2015). A molecular description of cellulose biosynthesis. Annu. Rev. Biochem..

[82] Torres F., Arroyo J., Troncoso O., Torres F., Arroyo J., Troncoso O., Torres F., Arroyo J., Troncoso O. (2019). Bacterial cellulose nanocomposites: An all-nano type of material. Mater. Sci. Eng. C.

[83] Dey D., Dharini V., Selvam S.P., Sadiku E.R., Kumar M.M., Jayaramudu J., Gupta U.N. (2021). Physical, antifungal, and biodegradable properties of cellulose nanocrystals and chitosan nanoparticles for food packaging application. Mater. Today Proc..

[84] Noshirvani N., Ghanbarzadeh B., Mokarram R.R., Hashemi M. (2017). Novel active packaging based on carboxymethyl cellulose-chitosan-ZnO NPs nanocomposite for increasing the shelf life of bread. Food Packag. Shelf Life.

[85] Bertoft E. (2017). Understanding starch structure: Recent progress. Agronomy.

[86] Cheng H., Chen L., McClements D.J., Yang T., Zhang Z., Ren F., Miao M., Tian Y., Jin Z. (2021). Starch-based biodegradable packaging materials: A review of their preparation, characterization and diverse applications in the food industry. Trends Food Sci. Technol..

[87] Moeini A., Mallardo S., Cimmino A., Poggetto G.D., Masi M., Di Biase M., van Reenen A., Lavermicocca P., Valerio F., Evidente A. (2019). Thermoplastic starch and bioactive chitosan sub-microparticle biocomposites: Antifungal and chemico-physical properties of the films. Carbohydr. Polym..

[88] Díaz-Galindo E.P., Nesic A., Bautista-Baños S., García O.D., Cabrera-Barjas G. (2020). Corn-starch-based materials incorporated with cinnamon oil emulsion: Physico-chemical characterization and biological activity. Foods.

[89] Jiménez-Gómez C.P., Cecilia J.A. (2020). Chitosan: A Natural Biopolymer with a Wide and Varied Range of Applications. Molecules.

[90] El Knidri H., Belaabed R., Addaou A., Laajeb A., Lahsini A. (2018). Extraction, chemical modification and characterization of chitin and chitosan. Int. J. Biol. Macromol..

[91] Oladzadabbasabadi N., Nafchi A.M., Ariffin F., Wijekoon M.J.O., Al-Hassan A., Dheyab M.A., Ghasemlou M. (2022). Recent advances in extraction, modification, and application of chitosan in packaging industry. Carbohydr. Polym..

[92] de Oca-Vásquez G.M., Esquivel-Alfaro M., Vega-Baudrit J.R., Jiménez-Villalta G., Romero-Arellano V.H., Sulbarán-Rangel B. (2023). Development of nanocomposite chitosan films with antimicrobial activity from agave bagasse and shrimp shells. J. Agric. Food Res..

[93] Singh S., Nwabor O.F., Syukri D.M., Voravuthikunchai S.P. (2021). Chitosan-poly(vinyl alcohol) intelligent films fortified with anthocyanins isolated from *Clitoria ternatea* and *Carissa carandas* for monitoring beverage freshness. Int. J. Biol. Macromol..

[94] E Agustin Y., Padmawijaya K.S. (2017). Effect of glycerol and zinc oxide addition on antibacterial activity of biodegradable bioplastics from chitosan-kepok banana peel starch. IOP Conf. Ser. Mater. Sci. Eng..

[95] Zhao Y., Li B., Li C., Xu Y., Luo Y., Liang D., Huang C. (2021). Comprehensive Review of Polysaccharide-Based Materials in Edible Packaging: A Sustainable Approach. Foods.

[96] Shen Y., Seidi F., Ahmad M., Liu Y., Saeb M.R., Akbari A., Xiao H. (2023). Recent Advances in Functional Cellulose-based Films with Antimicrobial and Antioxidant Properties for Food Packaging. J. Agric. Food Chem..

[97] Zainal S.H., Mohd N.H., Suhaili N., Anuar F.H., Lazim A.M., Othaman R. (2021). Preparation of cellulose-based hydrogel: A review. J. Mater. Res. Technol..

[98] Hassanloofard Z., Gharekhani M., Zandi M., Ganjloo A., Roufegarinejad L. (2023). Fabrication and characterization of cellulose acetate film containing Falcaria vulgaris extract. Cellulose.

[99] Hemraz U.D., Lam E., Sunasee R. (2023). Recent advances in cellulose nanocrystals-based antimicrobial agents. Carbohydr. Polym..

[100] Amoroso L., De France K.J., Kummer N., Ren Q., Siqueira G., Nyström G. (2023). Nanocomposites of cellulose nanofibers incorporated with carvacrol via stabilizing octenyl succinic anhydride-modified ε-polylysine. Int. J. Biol. Macromol..

[101] Bangar S.P., Esua O.J., Nickhil C., Whiteside W.S. (2023). Microcrystalline cellulose for active food packaging applications: A review. Food Packag. Shelf Life.

[102] Firmanda A., Fahma F., Warsiki E., Syamsu K., Arnata I.W., Sartika D., Suryanegara L., Qanytah, Suyanto A. (2023). Antimicrobial mechanism of nanocellulose composite packaging incorporated with essential oils. Food Control.

[103] Romainor A.N., Chin S.F., Lihan S. (2021). Antimicrobial Starch-Based Film for Food Packaging Application. Starch.

[104] Hashem A.H., El-Naggar M.E., Abdelaziz A.M., Hassan Y.R., Hasain M.S. (2023). Bio-based antimicrobial food packaging films based on hydroxypropyl starch/polyvinyl alcohol loaded with the biosynthesized zinc oxide nanoparticles. Int. J. Biol. Macromol..

[105] Muller J., González-Martínez C., Chiralt A. (2017). Combination of Poly(lactic) acid and starch for biodegradable food packaging. Materials.

[106] Liu J., Wang Y., Hou X., Cui Q., Wu H., Shen G., Luo Q., Li S., Liu X., Li M. (2023). Starch-based film functionalized with *Zanthoxylum armatum* essential oil improved the shelf life of beef sauce. LWT.

[107] Chen L., Wu F., Xiang M., Zhang W., Wu Q., Lu Y., Fu J., Chen M., Li S., Chen Y. (2023). Encapsulation of tea polyphenols into high amylose corn starch composite nanofibrous film for active antimicrobial packaging. Int. J. Biol. Macromol..

[108] Ardjoum N., Chibani N., Shankar S., Salmieri S., Djidjelli H., Lacroix M. (2023). Incorporation of Thymus vulgaris essential oil and ethanolic extract of propolis improved the antibacterial, barrier and mechanical properties of corn starch-based films. Int. J. Biol. Macromol..

[109] Bangar S.P., Whiteside W.S., Suri S., Barua S., Phimolsiripol Y. (2023). Native and modified biodegradable starch-based packaging for shelf-life extension and safety of fruits/vegetables. Int. J. Food Sci. Technol..

[110] Srivastava V., Singh S., Das D. (2023). Rice husk fiber-reinforced starch antimicrobial biocomposite film for active food packaging. J. Clean. Prod..

[111] Flórez M., Guerra-Rodriguez E., Cazón P., Vásquez M. (2022). Chitosan for food packaging: Recent advances in active and intelligent films. Food Hydrocoll..

[112] Younes I., Rinaudo M. (2015). Chitin and chitosan preparation from marine sources. Structure, properties and applications. Mar. Drugs.

[113] Stefanowska K., Woźniak M., Dobrucka R., Ratajczak I. (2023). Chitosan with Natural Additives as a Potential Food Packaging. Materials.

[114] Mesgari M., Aalami A.H., Sahebkar A. (2021). Antimicrobial activities of chitosan/titanium dioxide composites as a biological nanolayer for food preservation: A review. Int. J. Biol. Macromol..

[115] Quesada J., Sendra E., Navarro C., Sayas-Barberá E. (2016). Antimicrobial active packaging including chitosan films with *Thymus vulgaris* L. Essential oil for ready-to-eat meat. Foods.

[116] Zhang X., Xiao G., Wang Y., Zhao Y., Su H., Tan T. (2017). Preparation of chitosan-TiO_2_ composite film with efficient antimicrobial activities under visible light for food packaging applications. Carbohydr. Polym..

[117] Wang K., Lim P.N., Tong S.Y., Thian E.S. (2019). Development of grapefruit seed extract-loaded poly(ε-caprolactone)/chitosan films for antimicrobial food packaging. Food Packag. Shelf Life.

[118] Boura-Theodoridou O., Giannakas A., Katapodis P., Stamatis H., Ladavos A., Barkoula N.-M. (2020). Performance of ZnO/chitosan nanocomposite films for antimicrobial packaging applications as a function of NaOH treatment and glycerol/PVOH blending. Food Packag. Shelf Life.

[119] Casalini S., Baschetti M.G. (2023). The use of essential oils in chitosan or cellulose-based materials for the production of active food packaging solutions: A review. J. Sci. Food Agric..

[120] Babaei-Ghazvini A., Acharya B., Korber D.R. (2021). Antimicrobial biodegradable food packaging based on chitosan and metal/metal-oxide bio-nanocomposites: A review. Polymers.

[121] Muhammad A., Lee D., Shin Y., Park J. (2021). Recent progress in polysaccharide aerogels: Their synthesis, application, and future outlook. Polymers.

[122] Ganesan K., Budtova T., Ratke L., Gurikov P., Baudron V., Preibisch I., Niemeyer P., Smirnova I., Milow B. (2018). Review on the production of polysaccharide aerogel particles. Materials.

[123] Zhou W., Fang J., Tang S., Wu Z., Wang X. (2021). 3D-printed nanocellulose-based cushioning–antibacterial dual-function food packaging aerogel. Molecules.

[124] da Silva F.T., de Oliveira J.P., Fonseca L.M., Bruni G.P., da Rosa Zavareze E., Dias A.R.G. (2020). Physically cross-linked aerogels based on germinated and non-germinated wheat starch and PEO for application as water absorbers for food packaging. Int. J. Biol. Macromol..

[125] Kumari M., Giri V.P., Pandey S., Kumar M., Katiyar R., Nautiyal C.S., Mishra A. (2019). An insight into the mechanism of antifungal activity of biogenic nanoparticles than their chemical counterparts. Pestic. Biochem. Physiol..

[126] Harish V., Tewari D., Gaur M., Yadav A.B., Swaroop S., Bechelany M., Barhoum A. (2022). Review on Nanoparticles and Nanostructured Materials: Bioimaging, Biosensing, Drug Delivery, Tissue Engineering, Antimicrobial, and Agro-Food Applications. Nanomaterials.

[127] Khan I., Saeed K., Khan I. (2019). Nanoparticles: Properties, applications and toxicities. Arab. J. Chem..

[128] Sikder M., Wang J., Poulin B.A., Tfaily M.M., Baalousha M. (2020). Nanoparticle size and natural organic matter composition determine aggregation behavior of polyvinylpyrrolidone coated platinum nanoparticles. Environ. Sci. Nano.

[129] Abdel-Haleem F.M., Gamal E., Rizk M.S., Madbouly A., El Nashar R.M., Anis B., Elnabawy H.M., Khalil A.S.G., Barhoum A. (2021). Molecularly Imprinted Electrochemical Sensor-Based Fe_2_O_3_@MWCNTs for Ivabradine Drug Determination in Pharmaceutical Formulation, Serum, and Urine Samples. Front. Bioeng. Biotechnol..

[130] Arriagada F., Correa O., Günther G., Nonell S., Mura F., Olea-Azar C., Morales J. (2016). Morin flavonoid adsorbed on mesoporous silica, a novel antioxidant nanomaterial. PLoS ONE.

[131] Joseph A., Wood T., Chen C.-C., Corry K., Snyder J.M., Juul S.E., Parikh P., Nance E. (2018). Curcumin-loaded polymeric nanoparticles for neuroprotection in neonatal rats with hypoxic-ischemic encephalopathy. Nano Res..

[132] Medici S., Peana M., Pelucelli A., Zoroddu M.A. (2021). An updated overview on metal nanoparticles toxicity. Semin. Cancer Biol..

[133] Ashfaq A., Khursheed N., Fatima S., Anjum Z., Younis K. (2022). Application of nanotechnology in food packaging: Pros and Cons. J. Agric. Food Res..

[134] Gupta R.K., El Gawad F.A., Ali E.A., Karunanithi S., Yugiani P., Srivastav P.P. (2024). Nanotechnology: Current applications and future scope in food packaging systems. Meas. Food.

[135] Echegoyen Y., Nerín C. (2013). Nanoparticle release from nano-silver antimicrobial food containers. Food Chem. Toxicol..

[136] Mackevica A., Olsson M.E., Hansen S.F. (2016). Silver nanoparticle release from commercially available plastic food containers into food simulants. J. Nanoparticle Res..

[137] Ramos-Zúñiga J., Bruna N., Pérez-Donoso J.M. (2023). Toxicity Mechanisms of Copper Nanoparticles and Copper Surfaces on Bacterial Cells and Viruses. Int. J. Mol. Sci..

[138] Gonzalez-Estrella J., Puyol D., Gallagher S., Sierra-Alvarez R., Field J.A. (2015). Elemental copper nanoparticle toxicity to different trophic groups involved in anaerobic and anoxic wastewater treatment processes. Sci. Total Environ..

[139] More S.J., Bampidis V., Benford D., Bragard C., Halldorsson T.I., Hernández-Jerez A.F., Bennekou S.H., Koutsoumanis K., Lambré C., EFSA Scientific Committee (2023). Re-evaluation of the existing health-based guidance values for copper and exposure assessment from all sources. EFSA J..

[140] Cushen M., Kerry J., Morris M., Cruz-Romero M., Cummins E. (2013). Migration and exposure assessment of silver from a PVC nanocomposite. Food Chem..

[141] Slavin Y.N., Bach H. (2022). Mechanisms of Antifungal Properties of Metal Nanoparticles. Nanomaterials.

[142] Suryanegara L., Fatriasari W., Zulfiana D., Anita S.H., Masruchin N., Gutari S., Kemala T. (2021). Novel antimicrobial bioplastic based on PLA-chitosan by addition of TiO_2_ and ZnO. J. Environ. Health Sci. Eng..

[143] Garcia E.L., Attallah O.A., Mojicevic M., Devine D.M., Fournet M.B. (2021). Antimicrobial active bioplastics using triangular silver nanoplate integrated polycaprolactone and polylactic acid films. Materials.

[144] Kupka T., Buczek A., Broda M.A., Stachów M., Tarnowski P. (2016). DFT studies on the structural and vibrational properties of polyenes. J. Mol. Model..

[145] Tevyashova A., Efimova S., Alexandrov A., Omelchuk O., Ghazy E., Bychkova E., Zatonsky G., Grammatikova N., Dezhenkova L., Solovieva S. (2023). Semisynthetic Amides of Amphotericin B and Nystatin A_1_: A Comparative Study of In Vitro Activity/Toxicity Ratio in Relation to Selectivity to Ergosterol Membranes. Antibiotics.

[146] Pekmezovic M., Krusic M.K., Malagurski I., Milovanovic J., Stępień K., Guzik M., Charifou R., Babu R., O’cOnnor K., Nikodinovic-Runic J. (2021). Polyhydroxyalkanoate/antifungal polyene formulations with monomeric hydroxyalkanoic acids for improved antifungal efficiency. Antibiotics.

[147] Humelnicu A.-C., Samoilă P., Cojocaru C., Dumitriu R., Bostănaru A.-C., Mareș M., Harabagiu V., Simionescu B.C. (2022). Chitosan-Based Therapeutic Systems for Superficial Candidiasis Treatment. Synergetic Activity of Nystatin and Propolis. Polymers.

[148] Sen P., Vijay M., Singh S., Hameed S., Vijayaraghvan P. (2022). Understanding the environmental drivers of clinical azole resistance in Aspergillus species. Drug Target Insights.

[149] Shafiei M., Peyton L., Hashemzadeh M., Foroumadi A. (2020). History of the development of antifungal azoles: A review on structures, SAR, and mechanism of action. Bioorgan. Chem..

[150] Warrilow A.G.S., Martel C.M., Parker J.E., Melo N., Lamb D.C., Nes W.D., Kelly D.E., Kelly S.L. (2010). Azole binding properties of *Candida albicans* sterol 14-α demethylase (CaCYP51). Antimicrob. Agents Chemother..

[151] Bajunaid S.O. (2022). How Effective Are Antimicrobial Agents on Preventing the Adhesion of *Candida albicans* to Denture Base Acrylic Resin Materials? A Systematic Review. Polymers.

[152] Vlaia L., Olariu I., Muţ A.M., Coneac G., Vlaia V., Anghel D.F., Maxim M.E., Stângă G., Dobrescu A., Suciu M. (2022). New, Biocompatible, Chitosan-Gelled Microemulsions Based on Essential Oils and Sucrose Esters as Nanocarriers for Topical Delivery of Fluconazole. Pharmaceutics.

[153] Biesalski H.-K., Dragsted L.O., Elmadfa I., Grossklaus R., Müller M., Schrenk D., Walter P., Weber P. (2009). Bioactive compounds: Definition and assessment of activity. Nutrition.

[154] Christaki E., Bonos E., Giannenas I., Florou-Paneri P. (2012). Aromatic plants as a source of bioactive compounds. Agriculture.

[155] Altemimi A., Lakhssassi N., Baharlouei A., Watson D.G., Lightfoot D.A. (2017). Phytochemicals: Extraction, isolation, and identification of bioactive compounds from plant extracts. Plants.

[156] Abubakar A.R., Haque M. (2020). Preparation of medicinal plants: Basic extraction and fractionation procedures for experimental purposes. J. Pharm. Bioallied Sci..

[157] Azmir J., Zaidul I.S.M., Rahman M.M., Sharif K.M., Mohamed A., Sahena F., Jahurul M.H.A., Ghafoor K., Norulaini N.A.N., Omar A.K.M. (2013). Techniques for extraction of bioactive compounds from plant materials: A review. J. Food Eng..

[158] Aharoni A., Galili G. (2011). Metabolic engineering of the plant primary–secondary metabolism interface. Curr. Opin. Biotechnol..

[159] Atanasov A.G., Waltenberger B., Pferschy-Wenzig E.-M., Linder T., Wawrosch C., Uhrin P., Temml V., Wang L., Schwaiger S., Heiss E.H. (2015). Discovery and resupply of pharmacologically active plant-derived natural products: A review. Biotechnol. Adv..

[160] Rodino S., Butu M. (2019). Herbal extracts-new trends in functional and medicinal beverages. Functional and Medicinal Beverages: Volume 11: The Science of Beverages.

[161] Pott D.M., Osorio S., Vallarino J.G. (2019). From central to specialized metabolism: An overview of some secondary compounds Derived from the primary metabolism for their role in conferring nutritional and organoleptic characteristics to fruit. Front. Plant Sci..

[162] Twaij B.M., Hasan N. (2022). Bioactive Secondary Metabolites from Plant Sources: Types, Synthesis, and Their Therapeutic Uses. Int. J. Plant Biol..

[163] Römer S., Fraser P.D. (2005). Recent advances in carotenoid biosynthesis, regulation and manipulation. Planta.

[164] Yang L., Stöckigt J. (2010). Trends for diverse production strategies of plant medicinal alkaloids. Nat. Prod. Rep..

[165] Matsuura H.N., Fett-Neto A.G. (2015). Plant Alkaloids: Main Features, Toxicity, and Mechanisms of Action. Plant Toxins.

[166] Silvestre A.J.D., Gandini A. (2008). Terpenes: Major sources, properties and applications. Monomers, Polymers and Composites from Renewable Resources.

[167] Masyita A., Sari R.M., Astuti A.D., Yasir B., Rumata N.R., Emran T.B., Nainu F., Simal-Gandara J. (2022). Terpenes and terpenoids as main bioactive compounds of essential oils, their roles in human health and potential application as natural food preservatives. Food Chem. X.

[168] Zinno P., Guantario B., Lombardi G., Ranaldi G., Finamore A., Allegra S., Mammano M.M., Fascella G., Raffo A., Roselli M. (2023). Chemical Composition and Biological Activities of Essential Oils from *Origanum vulgare* Genotypes Belonging to the Carvacrol and Thymol Chemotypes. Plants.

[169] Rao A., Zhang Y.Q., Muend S., Rao R. (2010). Mechanism of antifungal activity of terpenoid phenols resembles calcium stress and inhibition of the TOR pathway. Antimicrob. Agents Chemother..

[170] Chaillot J., Tebbji F., Remmal A., Boone C., Brown G.W., Bellaoui M., Sellam A. (2015). The monoterpene carvacrol generates endoplasmic reticulum stress in the pathogenic fungus *Candida albicans*. Antimicrob. Agents Chemother..

[171] Papachristos D.P., I Karamanoli K., Stamopoulos D.C., Menkissoglu-Spiroudi U. (2004). The relationship between the chemical composition of three essential oils and their insecticidal activity against *Acanthoscelides obtectus* (Say). Pest Manag. Sci..

[172] Nóbrega J.R., Silva D.d.F., Júnior F.P.d.A., Sousa P.M.S., de Figueiredo P.T.R., Cordeiro L.V., Lima E.d.O. (2021). Antifungal action of α-pinene against *Candida* spp. isolated from patients with otomycosis and effects of its association with boric acid. Nat. Prod. Res..

[173] Andrade A.C.d.M., Rosalen P.L., Freires I.A., Scotti L., Scotti M.T., Aquino S.G., de Castro R.D. (2018). Antifungal Activity, Mode of Action, Docking Prediction and Anti-biofilm Effects of (+)-β-pinene Enantiomers against *Candida* spp.. Curr. Top. Med. Chem.

[174] Balahbib A., El Omari N., Hachlafi N.E., Lakhdar F., El Menyiy N., Salhi N., Mrabti H.N., Bakrim S., Zengin G., Bouyahya A. (2021). Health beneficial and pharmacological properties of p-cymene. Food Chem. Toxicol..

[175] Marchese A., Arciola C.R., Barbieri R., Silva A.S., Nabavi S.M., Sokeng A.J.T., Izadi M., Jafari N.J., Suntar I., Daglia M. (2017). Update on monoterpenes as antimicrobial agents: A particular focus on p-cymene. Materials.

[176] Tian F., Woo S.Y., Lee S.Y., Chun H.S. (2018). p-Cymene and its derivatives exhibit antiaflatoxigenic activities against Aspergillus flavus through multiple modes of action. Appl. Biol. Chem..

[177] Ultee A., Bennik M.H.J., Moezelaar R. (2002). The phenolic hydroxyl group of carvacrol is essential for action against the food-borne pathogen *Bacillus cereus*. Appl. Environ. Microbiol..

[178] Pinto L., Bonifacio M.A., De Giglio E., Cometa S., Logrieco A.F., Baruzzi F. (2020). Unravelling the antifungal effect of red thyme oil (*Thymus vulgaris* L.) compounds in vapor phase. Molecules.

[179] Yu D., Wang J., Shao X., Xu F., Wang H. (2015). Antifungal modes of action of tea tree oil and its two characteristic components against *Botrytis cinerea*. J. Appl. Microbiol..

[180] Nainu F., Masyita A., Bahar M.A., Raihan M., Prova S.R., Mitra S., Bin Emran T., Simal-Gandara J. (2021). Pharmaceutical prospects of bee products: Special focus on anticancer, antibacterial, antiviral, and antiparasitic properties. Antibiotics.

[181] Ożarowski M., Karpiński T.M., Alam R., Łochyńska M. (2022). Antifungal Properties of Chemically Defined Propolis from Various Geographical Regions. Microorganisms.

[182] Li Z., Liu M., Dawuti G., Dou Q., Ma Y., Liu H., Aibai S. (2017). Antifungal Activity of Gallic Acid In Vitro and In Vivo. Phytotherapy Res..

[183] de Morais M.C., Perez-Castillo Y., Silva V.R., Santos L.d.S., Soares M.B.P., Bezerra D.P., de Castro R.D., de Sousa D.P. (2021). Cytotoxic and antifungal amides derived from ferulic acid: Molecular docking and mechanism of action. BioMed Res. Int..

[184] Pernin A., Bosc V., Maillard M.-N., Dubois-Brissonnet F. (2019). Ferulic acid and eugenol have different abilities to maintain their inhibitory activity against *Listeria monocytogenes* in emulsified systems. Front. Microbiol..

[185] Ibitoye O., Ajiboye T. (2019). Ferulic acid potentiates the antibacterial activity of quinolone-based antibiotics against *Acinetobacter baumannii*. Microb. Pathog..

[186] Pietta P., Minoggio M., Bramati L. (2003). Plant polyphenols: Structure, occurrence and bioactivity. Studies in Natural Products Chemistry.

[187] Qian W., Yang M., Wang T., Sun Z., Liu M., Zha J., Zeng Q., Cai C., Li Y. (2020). Antibacterial Mechanism of Vanillic Acid on Physiological, Morphological, and Biofilm Properties of Carbapenem-Resistant *Enterobacter hormaechei*. J. Food Prot..

[188] Nazzaro F., Fratianni F., Coppola R., De Feo V. (2017). Essential oils and antifungal activity. Pharmaceuticals.

[189] Sadgrove N.J., Padilla-González G.F., Phumthum M. (2022). Fundamental Chemistry of Essential Oils and Volatile Organic Compounds, Methods of Analysis and Authentication. Plants.

[190] Brahmi F., Mokhtari O., Yahyaoui M.I., Zraibi L., Bentouhami N.E., Abdeslam A., Legssyer B. (2023). Phytochemical composition, antioxidant, and antifungal activity of essential oil from *Myrtus communis*, L.. Mater. Today Proc..

[191] Yan J., Wu H., Chen K., Feng J., Zhang Y. (2021). Antifungal activities and mode of action of *Cymbopogon citratus*, *Thymus vulgraris*, and *Origanum heracleoticum* essential oil vapors against *Botrytis cinerea* and their potential application to control postharvest strawberry gray mold. Foods.

[192] Yan J., Wu H., Shi F., Wang H., Chen K., Feng J., Jia W. (2021). Antifungal activity screening for mint and thyme essential oils against *Rhizopus stolonifer* and their application in postharvest preservation of strawberry and peach fruits. J. Appl. Microbiol..

[193] Xu J., Shao X., Li Y., Wei Y., Xu F., Wang H. (2017). Metabolomic analysis and mode of action of metabolites of tea tree oil involved in the suppression of botrytis cinerea. Front. Microbiol..

[194] Shao X., Cheng S., Wang H., Yu D., Mungai C. (2013). The possible mechanism of antifungal action of tea tree oil on *Botrytis cinerea*. J. Appl. Microbiol..

[195] Allagui M.B., Moumni M., Romanazzi G. (2024). Antifungal Activity of Thirty Essential Oils to Control Pathogenic Fungi of Postharvest Decay. Antibiotics.

[196] Schiavon G., Garello M., Prencipe S., Meloni G.R., Buonsenso F., Spadaro D. (2023). Essential Oils Reduce Grey Mould Rot of Apples and Modify the Fruit Microbiome during Postharvest Storage. J. Fungi.

[197] Álvarez-García S., Moumni M., Romanazzi G. (2023). Antifungal activity of volatile organic compounds from essential oils against the postharvest pathogens *Botrytis cinerea*, *Monilinia fructicola*, *Monilinia fructigena*, and *Monilinia laxa*. Front. Plant Sci..

[198] De Souza V.V.M.A., Crippa B.L., De Almeida J.M., Iacuzio R., Setzer W.N., Sharifi-Rad J., Silva N.C.C. (2020). Synergistic antimicrobial action and effect of active chitosan-gelatin biopolymeric films containing *Thymus vulgaris*, *Ocimum basilicum* and *Origanum majorana* essential oils against *Escherichia coli* and *Staphylococcus aureus*. Cell. Mol. Biol..

[199] Sharma N., Tripathi A. (2008). Effects of *Citrus sinensis* (L.) Osbeck epicarp essential oil on growth and morphogenesis of *Aspergillus niger* (L.) Van Tieghem. Microbiol. Res..

